# Comparison of Mucosal, Subcutaneous and Intraperitoneal Routes of Rat *Leptospira* Infection

**DOI:** 10.1371/journal.pntd.0004569

**Published:** 2016-03-31

**Authors:** Anne-Laure Zilber, Patrick Belli, Delphine Grezel, Marc Artois, Angeli Kodjo, Zoheira Djelouadji

**Affiliations:** 1 USC 1233 INRA/VAS, Equipe de Recherche sur la Leptospirose, Université de Lyon-VetAgro Sup, Marcy-l’Etoile, France; 2 UP Pathologie Morphologique et Clinique, Université de Lyon-VetAgro Sup, Marcy-l’Etoile, France; 3 Université de Lyon-VetAgro Sup, Marcy-l’Etoile, France; 4 Laboratoire des Leptospires, Université de Lyon-VetAgro Sup, Marcy-l’Etoile, France; University of California San Diego School of Medicine, UNITED STATES

## Abstract

Leptospirosis is a zoonosis found worldwide that is caused by a spirochete. The main reservoirs of *Leptospira*, which presents an asymptomatic infection, are wild rodents, including the brown rat (*Rattus norvegicus*). Experimental studies of the mechanisms of its renal colonization in rats have previously used an intraperitoneal inoculation route. However, knowledge of rat-rat transmission requires the use of a natural route of inoculation, such as a mucosal or subcutaneous route. We investigated for the first time the effects of subcutaneous and mucosal inoculation routes compared to the reference intraperitoneal route during *Leptospira* infection in adult rats. Infection characteristics were studied using *Leptospira* renal isolation, serology, and molecular and histological analyses. *Leptospira* infection was asymptomatic using each inoculation route, and caused similar antibody production regardless of renal colonization. The observed renal colonization rates were 8 out of 8 rats, 5 out of 8 rats and 1 out of 8 rats for the intraperitoneal, mucosal and subcutaneous inoculation routes, respectively. Thus, among the natural infection routes studied, mucosal inoculation was more efficient for renal colonization associated with urinary excretion than the subcutaneous route and induced a slower-progressing infection than the intraperitoneal route. These results can facilitate understanding of the infection modalities in rats, unlike the epidemiological studies conducted in wild rats. Future studies of other natural inoculation routes in rat models will increase our knowledge of rat-rat disease transmission and allow the investigation of infection kinetics.

## Author Summary

Leptospirosis (infection with pathogenic *Leptospira* spp.) is a public health concern worldwide. The brown rat (*Rattus norvegicus*), as the most ubiquitous animal of urban wildlife, is potentially the primary source of *Leptospira* spp. for humans, dogs and livestock. For understanding the *Leptospira* maintenance in rat colonies, the experimental studies required the use of natural route of transmission between the rats. We investigated the effects of the mucosal and bite’s transmission (conjunctival-mucosal and subcutaneous routes) compared to the reference route (intraperitoneal) during infection in adult rats. With serology, we showed that the antibody production was independent of the inoculation route. By isolation, molecular and histological analyses, we found that the mucosal route was more efficient at renal colonization and leptospires excretion than the subcutaneous route. These results can be useful in understanding the infection modalities in rat that could prevent the human leptospirosis.

## Introduction

Leptospirosis is a worldwide zoonosis that is caused by a spirochete of the genus *Leptospira* [1]. The World Health Organization (WHO) reports one million severe human cases of leptospirosis each year [2,3], especially in tropical and subtropical regions. Recently, the number of reported outbreaks has increased with rainfall and urbanization associated with the conditions of slum living [4,5]. Human leptospirosis ranges from a mild form to a severe infection called Weil’s disease, which has a fatality rate of 5–15% and is characterized by jaundice, renal failure and hemorrhage [6]. In animals, leptospirosis also causes reproductive failure, abortion and infertility in cattle [1], and acute febrile illness with renal and hepatic failure in dogs [7]. The main reservoirs of *Leptospira* are wild rodents, including the brown rat (*Rattus norvegicus*) [1,8]. The infection is asymptomatic in this animal, and leptospires persist by renal carriage, colonizing the proximal tubules [9]. Leptospires are secreted in the rat’s urine and infect humans and other animals by direct or indirect contact with the contaminated environment [5,10]. *Rattus* species appear to have a specific association with the Icterohaemorrhagiae serogroup [4,11] that causes most human cases of leptospirosis worldwide [2,11]. The control of the contamination of humans and domestic animals is therefore required in a rodent infection study.

The experimental approach used to study leptospiral infection often focuses on the mechanisms of pathogenicity, especially in the acute dose-response model of infection in guinea pigs or hamsters [12]. Rat models have been developed to study the mechanisms of *Leptospira* renal colonization or urinary excretion during chronic infection [13], but infection kinetics in rats are rarely studied. The intraperitoneal route of infection has been used in all rat studies [14–17], but this route could overestimate the dissemination time and the pathogen load during dissemination [14]. Moreover, the intraperitoneal route is a non-natural *Leptospira* infection route in rat colonies, and the details of transmission between rats remains unknown [18,19]. Studies of natural routes of rat-rat transmission could explain the heterogeneity of renal carriage in rat colonies from the same region [20,21] by variable *Leptospira* dissemination kinetics. Natural routes of infection, such as conjunctival, subcutaneous, epicutaneous and intradermal routes, have only been studied in acute infection models in guinea pigs and hamsters [22–24]. The kinetics of *Leptospira* dissemination exhibit significant variation depending on the inoculation route used. For example, abraded skin is a less efficient barrier to leptospires than intact skin [22]; in the same way, it has been shown that the conjunctival route requires a higher dose to cause lethality than do the subcutaneous and intraperitoneal routes [24]. The influence of a natural inoculation route remains to be studied in rat models. The conjunctival-mucosal and subcutaneous inoculation routes are natural routes of rat transmission. The conjunctival-mucosal route corresponds to mucosal transmission by environmental contamination, and the subcutaneous route corresponds to direct contamination from a rat bite [21], according to the most recent hypothesis regarding transmission between rats via the saliva and biting [19,21]. Both routes could significantly affect dissemination time and renal colonization.

In this study, we investigated for the time the establishment of a rat infection model based on natural disease transmission routes in rat colonies. The objectives of this study were as follows: 1) to report the effect of subcutaneous and mucosal inoculation routes on the renal colonization and urinary excretion of *Leptospira* infection compared to the reference intraperitoneal inoculation route; and 2) to investigate others potential excretion routes such as saliva or feces.

## Materials and Methods

### Leptospiral strain used for rat inoculation

A virulent *Leptospira interrogans* serovar Copenhageni strain Fiocruz L1-130, provided by the National Reference Center and WHO collaboration Center for Leptospirosis (Institut Pasteur, Paris, France), was used in all experiments. This same strain has been used in several experimental studies in rats [14,25], is a clinical isolate from Brazil [26]. Leptospires were cultivated in Ellinghausen-McCullough-Johnson-Harris (EMJH) media (Indicia, Sainte-Foy-l’Argentière, France) at 29°C.

Before the use of the obtained experimental strain in rats, a virulence test was performed using 8-week-old male Mongolian gerbils (Janvier Labs, France) intraperitoneally injected with *Leptospira* suspensions. The strain was found to be 100% lethal in the gerbil model even at a single dose of 10^1^ leptospires. Blood was collected by cardiac puncture after the humane killing of sick bacteremic animals on day 5 after inoculation and was aseptically transferred into tubes containing EMJH media (Indicia). Second-passage pathogenic cultures, derived from the cardiac puncture culture, were used for all experimental rat infections.

### Experimental infection

Specific pathogen-free 7-week-old male Wistar rats (*Rattus norvegicus*, RjHan:WI) (Janvier Labs) weighing 275 g were provided with food and drink ad libitum and housed in individual cages with specific enrichment in the form of nesting materials and wood chew sticks, which allowed both the absence of inter-individual contamination and the maintenance of normal conditions and behavior during the one-week acclimation period and one-month study duration. All rats were weighed and examined weekly over the course of one month for clinical and behavioral signs.

The experimental procedures encompassed the inoculation procedure, the sample collection and the final sample set. Groups of eight animals were infected with the *Leptospira* inoculum at day 0, either intraperitoneally (IP injection, 500 μL of EMJH containing 1×10^7^ leptospires), subcutaneously (SC injection, 500 μL of EMJH containing 1×10^7^ leptospires in the lumbar region) or conjunctival-mucosally (M, 100 μL of EMJH containing 0.5×10^7^ leptospires, dispensed as: 1) 25 μL on the left eye, 2) 25 μL in the left nostril and 3) 50 μL in the mouth). An inoculum of 1×10^7^ leptospires resulted in 100% infected animals (14). To standardize and validate the inoculum dose, the subcutaneous inoculation route was checked manually after injection, and the rats were habituated by training to accept a conjunctival-mucosal inoculation. Furthermore, a control group of two uninfected animals was established for each inoculation route, which received sterile EMJH distributed in the same manner as the corresponding inoculation.

Sample collection was performed under isoflurane anesthesia, as shown in the timeline of Table 1. Blood and/or serum samples (500 μL) were collected, using a 26 gauge needle, from the caudal vein. Urine samples were collected in a sterile aluminum box from vigil rats (on day 10) or from anesthetized rats (on other days) by sphincter relaxation. Feces were collected in a sterile aluminum box by spontaneous dropping. Saliva samples were collected by oral plugging with a cotton swab. On day 30, rats were anesthetized with isoflurane and sacrificed by cervical dislocation. At necropsy, samples were collected aseptically for serology, *Leptospira* isolation, and molecular and histological analyses. Blood (from an intracardiac puncture), urine, feces and saliva were collected. Bronchoalveolar lavage (BAL) fluid was collected from the lungs by tracheal catheterization with a 14 gauge needle followed by an injection with 5 mL of sterile phosphate-buffered saline (PBS) (Sigma-Aldrich France, Lyon, France). The kidneys, liver, lungs, spleen and salivary glands were also collected. The left eye was collected from all rats in the infected and control M groups.

**Table 1:** Sample collection of selected organs and excretions analyzed by qPCR for the *Lfb1* gene, micro-agglutination test and histology.

Day after inoculation	MAT	Selected organs or fluids for qPCR ^a^	Histology	Animal group ^b^
1	Serum	B	NA	Infected, Control
7	Serum	U	NA	Infected, Control
10	NA	U	NA	Infected
14	Serum	U, F	NA	Infected, Control
20	Serum	U, F, S	NA	Infected
25	NA	U, F, S	NA	Infected
30	Serum	B, U, F, S, K, Li, Sp, Lu, Sg, BAL	K, Li, Sp, Lu, Sg	Infected, Control

MAT: Micro-agglutination test.

^a^ qPCR performed on the *Lfb1* gene [28].

^b^ From the infected group, 8 rat samples were collected; from the control group, 2 rat samples were collected.

B: Blood; U: Urine; F: Feces; S: Saliva; K: Kidney; Li: Liver; Sp: Spleen; Lu: Lung; Sg: Salivary gland; BAL: Bronchoalveolar Lavage.

NA: not applicable

### Micro-agglutination test

Serological tests using the microscopic agglutination test (MAT) using WHO endpoints [27] were performed in the Laboratoire des Leptospires (VetAgro Sup, Marcy l’Etoile, France) using a panel of four *Leptospira interrogans* strains from the Icterohaemorrhagiae serogroup as antigens: serovar Copenhageni strain M20, serovar Copenhageni strain Fiocruz, serovar Icterohaemorrhagiae strain RGA and serovar Icterohaemorrhagiae strain Verdun. Blood samples were centrifuged for 5 minutes at 3500 rpm, and screening was performed with serum dilutions ranging from 1:50 to 1:6400. The analysis of each serum sample from each rat was repeated in triplicate.

### DNA extraction and quantitative PCR (qPCR)

The kidney, liver, spleen, lung, ocular and salivary gland samples were ground aseptically. A small amount (25 mg) was incubated with 180 μL ATL Buffer (Qiagen, Courtaboeuf, France) and 25 μL proteinase K for 2 hours. For the urine, blood, saliva and BAL matrices, 200 μL of each sample was lysed for 15 minutes. After proteinase K treatment, DNA was extracted from 200 μl of lysed tissue or 200 μl of lysed matrices using the QIAamp DNA mini kit (Qiagen) following the manufacturer’s instructions. DNA from fecal samples (200 mg) was extracted using the QIAmp DNA stool kit (Qiagen) following the manufacturer’s instructions. All DNA concentrations were controlled spectrophotometrically by measurement of the absorbance at 260/280 using a NanoDrop 2000 (Thermo Fischer Scientific, Illkirch, France). All DNA samples were stored at -20°C.

DNA was tested by qPCR SYBR Green using the Rotor-Gene Q (Qiagen). A qPCR primer pair amplified the *Lfb1* gene, as previously described by Merien et al. [28] and used in real-time PCR test [29]. qPCR was performed on a final volume of 25 μl containing 12 μl of Rotor-Gene SYBR Green PCR kit (Qiagen), 1 μl of forward primer (10 μM), 1 μl of reverse primer (10 μM), 6 μl of H_2_O and 5 μl of target DNA. Control reactions without target DNA were included in each assay. The following thermocycling program was used: initial incubation step at 95°C for 5 min, followed by 45 cycles of 95°C for 10 s and 61°C for 40 s. Standard curves were generated from bacterial suspensions containing 1×10^7^ *L*. *interrogans* Copenhageni Fiocruz/200 μL of DNA extract. Ten-fold serial dilutions from 10^7^ to 10^1^ leptospires were performed in TE Buffer (Ambion Life Technologies, Saint Aubin, France). From the stock solution, 1 μL of target DNA contained 1.25×10^6^ genomic copies based on the genome size of *L*. *interrogans* L1-130 (4.6 Mb): 1 genome is ~ 5 fg of genomic DNA. Each DNA sample was tested in duplicate or triplicate if variable results were observed. The Tm of each positive DNA sample was controlled with the Tm of the *L*. *interrogans* Copenhageni Fiocruz used as a reference.

### *Leptospira* renal isolation

Half of a kidney from each rat in the infected and control groups was crushed and aseptically transferred into tubes containing EMJH media (Indicia). A series of three dilution tubes were incubated at 29°C according to the protocol for pathogenic *Leptospira* isolation [30]. The tubes were examined weekly for three months using a dark-field microscope.

### Histological analysis

A sample of each organ (kidney, liver, spleen, lung and salivary gland) from each rat was fixed in 10% formaldehyde for 24 h and subsequently transferred to 70° ethanol. The tissues were embedded in paraffin and cut into 3 μm sections. For each rat, all the organ samples were embedded in the same paraffin block. One section of each block was then stained with hematoxylin-phloxine (HP) to observe morphological lesions. Two stains were used for the visualization of leptospires. For each rat, one section was stained with Warthin-Starry silver staining (Merck KGaA, Darmstadt, Germany) [31], and another section was subjected to immunohistochemistry with antiserum specific to the *L*. *interrogans* Icterohaemorrhagiae serovar. Positive control sections from one *Leptospira* carrier wild rat were included in each silver staining and immunohistochemistry assay. For immunohistochemistry, paraffin was removed from the sections with xylene and ethanol. The tissues were incubated in citrate buffer (pH = 6) for 1 h at 95°C and subsequently treated with 0.3% hydrogen peroxide for 10 min at room temperature. Nonspecific staining was blocked by incubation of the sections with Super Block (UltraTek HRP Anti-Polyvalent Lab Pack, ScyTek Laboratories, Logan, USA) for 30 min at room temperature, and rodent-specific sites were blocked by incubation of a 1,000-fold dilution of peroxidase-conjugated goat anti-rat antibody (Jackson ImmunoResearch Laboratories, West Grove, USA) for 10 min at room temperature. Tissue sections were incubated with a 2,000-fold dilution of *Leptospira* antiserum overnight at 4°C. The samples were then incubated with a 1:2 dilution of Ultra Tek Anti-Polyvalent (UltraTek HRP Anti-Polyvalent Lab Pack, ScyTek Laboratories) for 30 min at room temperature; subsequently, they were incubated with UltraTek HRP (UltraTek HRP Anti-Polyvalent Lab Pack, ScyTek Laboratories) at room temperature for 30 min. Enzymatic reactions were developed using the Vector NovaRED substrate kit for peroxidase (Vector Laboratories, Burlingame, USA). As an appropriate negative control, sections were incubated without *Leptospira* antiserum.

### Statistics

Bias reduction was achieved by a random allocation of rats in each group. Rats were taken in the same order for the collection of each sample.

The results are expressed as the median for the MAT and as the confidence interval for qPCR. Statistical tests were not used when fewer than 5 rats tested positive for leptospires. Bartlett’s one-way and permutational analysis of variance (ANOVA) test was used to examine the differences between multiple groups. Fisher’s test was applied to evaluate independence between multiple distributions. All statistical analyses were conducted using R software, version 2.15.2 (R Development Core Team, R foundation for statistical computing, Vienna, Austria).

### Animal ethics

All experimental procedures were performed according to the ethical and regulatory standards of the European Union Legislation governing the care and use of laboratory animals (Directive EU 10/63). All animal procedures were approved by the ethical committee of VetAgro Sup establishment (n°1288 for the gerbil procedure and n°1289 for the rat procedure) and were conducted by an authorized person (agreement no. 69–127811 issued by the Préfecture of the Rhône).

## Results

### Clinical response to infection and effect on body weight

The weekly general examinations of all infected and uninfected rats were normal throughout the study, without any clinical signs of infection observed.

The body weights of infected and control rats showed weight gains in the infected and control groups (between 129 g and 165 g, data are shown in S1 Table), corresponding to the expected growth of the 7-week-old rats during this month. The weight gain differences were not significant (p-value > 0.05) in the three infected groups, in the control groups and between the infected and control animals that were inoculated identically.

### Antibody response

**Fig 1:**
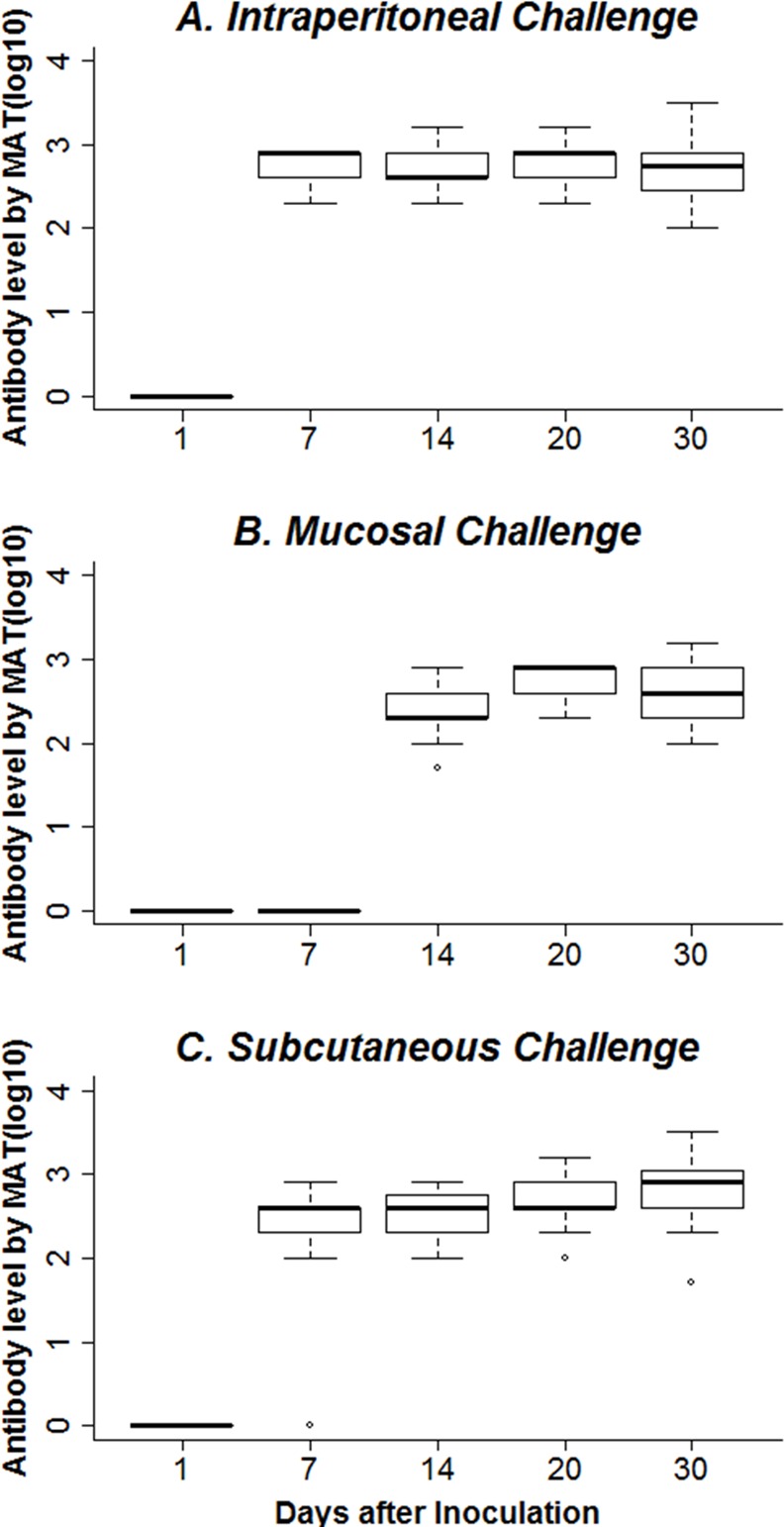
Antibody response in infected rats. Rat anti-*Leptospira* antibody responses were measured by the micro-agglutination test. The box plot shows the log10 titers of the sera samples of eight rats from intraperitoneal (A), mucosal (B) and subcutaneous (C) groups (tested in triplicate at a starting dilution of 1:50). Leptospiral antibody levels in each group were only significantly different on day 7 (p-value < 0.05).

All inoculated rats developed antibodies, and all the control animals remained negative during the study. A positive MAT titer was detected from day 7 post-inoculation for the 8 rats in the infected IP and SC groups and from day 14 for the M group (Fig 1). The antibody levels increased rapidly and appeared to stabilize after one week. The means of the MAT titers at day 30 were 701, 570 and 416 for the SC, IP and M groups, respectively. The titers of each rat in every infected group were similar irrespective of the results of the other methods. The leptospiral antibody levels of each group were significantly different at day 7 (p-value < 0.05), but the differences between the titers of the infected groups were not significant from day 14 until the end of the study (p-value > 0.05).

### Leptospiral organ and matrix burden analysis by qPCR

Organ and fluid samples were collected on days 1, 7, 10, 14, 20, 25 and 30 post-inoculation, as indicated in Table 1. Data obtained from the three groups of infected rats from day 7 to day 25 are summarized in Table 2, and all data from infected rats obtained at necropsy on day 30 are shown in Table 3 (The individual rat data are shown in S2 Table). All organ and matrix samples from control rats had negative qPCR results for the duration of the study.

**Table 2:** Results of blood, urine, feces and saliva samples from each infected group, analyzed by qPCR for the *Lfb1* gene on days 1 to 25.

	Intraperitoneal				Subcutaneous				Mucosal			
	Blood	Urine	Feces	Saliva	Blood	Urine	Feces	Saliva	Blood	Urine	Feces	Saliva
**Day 1**	8/8^a^ [109.45, 233.09] ^b^	ND	ND	ND	1/8 (9.15) ^c^	ND	ND	ND	0/8 (0)	ND	ND	ND
**Day 7**	ND	1/6 (135.87)	ND	ND	ND	0/7 (0)	ND	ND	ND	0/6 (0)	ND	ND
**Day 10**	ND	1/2 (543.75)	ND	ND	ND	0/3 (0)	ND	ND	ND	0/2 (0)	ND	ND
**Day 14**	ND	6/7 [10.63, 10843.75]	0/6 (0)	ND	ND	0/7 (0)	0/5 (0)	ND	ND	1/8 (147.58)	0/5 (0)	ND
**Day 20**	ND	6/7 [103.31, 4693.75]	0/6 (0)	0/8 (0)	ND	0/8 (0)	0/6 (0)	0/8 (0)	ND	0/8 (0)	0/3 (0)	0/8 (0)
**Day 25**	ND	5/7 [45.25, 2758.75]	0/6 (0)	0/8 (0)	ND	1/6 (57.50)	0/3 (0)	0/8 (0)	ND	2/8 [54.87, 1071.87]*	0/0 (0)	0/8 (0)

^a^: Number of positive samples/total number of rats in which samples could be collected.

^b^: The confidence intervals of the concentrations of genomic copies/μL of the positive samples with 0.95 probability are in brackets

* with 0.5 probability.

^c^: The concentration of genomic copies/μL of one positive sample is in parentheses.

ND: not determined

qPCR was performed on the *Lfb1* gene [28].

**Table 3:** Renal colonization and urinary excretions of each infected group on day 30.

	Urine qPCR	Kidney qPCR	Kidney isolation
**Intraperitoneal**	8/8 ^a^ [664.37, 3564.68] ^b^	8/8 [8.34, 93.69]	7/8
**Subcutaneous**	0/8 (0)	0/8 (0)	1/8
**Mucosal**	5/8 [120.21, 715]	3/8 [2.44, 11.58] *	4/8

^a^: Number of positives samples/total number of rats

^b^: The confidence intervals of the concentrations of genomic copies/μL of the positive samples with 0.95 probability are in brackets

*with 0.75 probability.

The other samples (blood, feces, saliva, liver, spleen, lung, salivary gland and bronchoalveolar lavage) tested negative for the *Lfb1* gene by qPCR [28].

On day 1, blood samples from the infected rats were positive in 8 of 8 rats from the IP group and in 1 of 8 rats from the SC group. In the M group, all 8 rats had negative blood samples. The numbers of genomic copies in the blood samples of rats from the infected groups are shown in Table 2.

*Leptospira* urinary excretion was examined using molecular analyses of urine samples collected every five days during the study. The results are shown in Table 2 and Table 3. On day 7, the urine sample from one rat in the IP group was positive, and no samples from rats in any other groups tested positive. A similar pattern was found on day 10, with one positive rat in the IP group but none in the other groups. For the IP infected group, 6 rats, 6 rats and 5 rats tested positive for leptospires on days 14, 20 and 25, respectively. Finally, on day 30, 8 out of 8 rats tested positive for leptospires. In the SC infected group, none of the 8 rats tested positive for leptospires before day 25 post-inoculation. On day 25, one rat tested positive for leptospires. However, on day 30, no rats tested positive. In the M infected group, one rat tested positive for leptospires on day 14. However, none of the 8 rats tested positive for leptospires on day 20. Finally, 2 rats and 5 out of 8 rats tested positive for leptospires on days 25 and day 30, respectively. All fecal samples and saliva matrices from the infected rats, collected from days 14 to 25, were negative (Table 2).

On day 30, all DNA samples from the blood, feces, saliva and BAL matrices and from the liver, spleen, lung and salivary gland tissues were negative. The DNA from the left eyes of the M group collected on day 30 was negative. Molecular analyses of DNA from the kidneys of infected rats revealed that 8 out of 8 rats tested positive in the IP group, whereas 0 of 8 rats from the SC group and 3 out of 8 rats from the M group tested positive for leptospires. All data from the renal molecular analyses are shown in Table 3.

### *Leptospira* renal isolation

Renal colonization was studied by *Leptospira* isolation from the kidney samples of all rats in both the infected and control groups. In the infected groups, a *Leptospira* culture was obtained in 7 out of 8 rats from the IP group, 4 out of 8 rats from the M group and 1 out of 8 rats from the SC group (Table 3), which are the same rats that tested positive by qPCR. All 6 rats from the control groups had negative renal cultures. Fisher’s test showed that the rate of culture corresponding to colonization was dependent on the inoculation route (p-value < 0.05).

### Histological analysis

Histological stains (HP, Warthin-Starry silver staining and immunohistochemistry) were conducted on sections of the kidney, liver, lung, spleen and salivary gland tissues from all rats in both the infected and control groups.

The HP staining revealed no lesions in the liver, spleen or salivary glands of all rats in the three infected groups. One pulmonary lesion was observed: perivascular lymphoid hypertrophy (data are shown in S1 Fig.). This lesion was observed in the infected rats (one rat in the SC group and two rats in the IP group). HP staining of the kidney sections showed that only 1 out of 8 rats in the SC group had interstitial nephritis (data are shown in S1 Fig.), although this rat tested negative for leptospires using PCR and isolation analyses. The other 7 rats from the SC group and all 8 rats from the IP and M groups, as well as those in the control groups, had no renal lesions.

No leptospires were observed in the liver, spleen, lung and salivary glands of any rats in the three infected groups or in the control groups by silver staining or immunohistochemistry. Additionally, silver staining and immunohistochemistry revealed that no *Leptospira* were found in the tubules of renal sections from the 24 infected rats and the 6 control rats. This observation may be due to the low detection sensitivity of histological method [32], together with the early phase of renal colonization characterized by small numbers of leptospires in the tubules.

## Discussion

We have studied for the first time a rat model for *Leptospira* infection based on the potential natural transmission routes of leptospirosis in rat colonies. We chose to investigate the conjunctival-mucosal and subcutaneous routes because they were shown to be efficient routes of infection in previous investigations of acute infection in susceptible species [23,24]. We report the effect of the inoculation route on *Leptospira* asymptomatic infection in a rat model and the results of investigations of other potential leptospire excretion routes.

### Asymptomatic infection and the effect of the inoculation route

Infections appeared to be identically asymptomatic regardless of the inoculation route (M, SC and IP). Indeed, the infections had no effect on weight gain or on clinical and behavioral examinations conducted during the study. This absence of weight loss in the adult rats is different than observations of weight loss in young rats [33] because they are not susceptible. Adult rats that carry *Leptospira* do not appear physically disadvantaged in rat colonies. Consequently, a carrier rat could be as healthy as a non-carrier rat.

Regarding serology, MAT titers were similar at the end of the month in all 3 groups. However, antibodies were detectable at day 7 post-inoculation for the SC and IP routes, while antibodies were only detectable at day 14 by the M route. The titers in chronic infections were considerably lower than the titers reported in acute infections, and no relationship was found between antibody production and renal colonization rate. This similar observation in field rats may result from an adaptation between the host and the pathogen [34] and may create a bias for MAT epidemiological studies. Hence, MAT only indicates exposure to *Leptospira* of animals suspected of carrying leptospires.

In this study, leptospiral bacteremia differed based on the inoculation route. Using molecular analysis, bacteremia was detected on day 1 for the SC and IP routes but not for the M route. The absence of bacteremia at day 1 and the detection of an MAT titer by day 14 for the M route may be a result of slower bacterial spread, likely delayed by passing the conjunctival and mucosal barrier.

The renal colonization rate varied significantly depending on the inoculation route. All rats in the IP infected group were renal carriers. Our IP rate was similar to rates observed in other experimental rat model studies, in which 100% of rats were densely colonized after one month of inoculation [14,16], but this inoculation route was not natural. Consistent with this result, the rats in our study that were inoculated via the SC and the M routes had renal colonization rates of 1 out of 8 and 5 out of 8, respectively, as shown and defined by positive results for molecular analyses of kidney and urine together with renal isolation. The sensitivity of the isolation and molecular, serological and histological methods was less than 100% [32,35], and thus it was necessary to combine several types of methods to obtain a reliable reflection of renal *Leptospira* colonization in infected rats. The decreased sensitivity of each method can be explained by small numbers of *Leptospira* in the renal tubule samples obtained during the samples collection in the dissection, resulting in bias [29]. The natural M inoculation route allowed a higher rate of renal colonization than the SC inoculation route.

In histological analysis, HP sections from only one rat in the SC group showed interstitial nephritis in the kidney, although this rat was negative for leptospires by PCR and by isolation. Interstitial nephritis is the only lesion attributable to leptospiral infection in rats [25], but in our study, renal carrier rats had no lesions. This absence of lesions may be the result of observing only one section for each staining assay that did not contain leptospires, although all collected organs from all rats in both infected and control groups have been observed.

Urinary excretion in rats infected by the IP route began on days 7–10, as observed in other studies [14,16], but leptospires were not detected in the urinary excretion of rats from the SC and M groups until day 25. Therefore, the natural infection routes affected both the time course of *Leptospira* dissemination in the body and the renal colonization associated with urinary excretion.

### *Leptospira* transmission in rat colonies

We investigated the potential natural transmission routes of *Leptospira*, the conjunctival-mucosal and the subcutaneous routes, in rat colonies by studying the kinetics of infection and excretion of leptospires from rats. The conjunctival-mucosal inoculation route corresponds to mucosal transmission from environmental contamination, such as drinking from a contaminated water source and splashing water into the eyes or nose. The subcutaneous route mimics transmission from a rat bite [21], with contamination of the wound by *Leptospira*. Our study showed that the mucosal route was more efficient at renal colonization associated with *Leptospira* urinary excretion than the subcutaneous route. In our study, the M inoculum was 2-fold less concentrated than the SC inoculum because of technical constraints, and in acute infections, the M inoculation route required a higher lethal dose [24]. Moreover, the mucosal route induced a slower progression of chronic infection. Previous studies using the intraperitoneal inoculation route created a bias for dissemination time and infection progression. Therefore, rat-rat transmission could require a high dose of *Leptospira* [14], but the transmission route is also important and significantly affects the renal colonization rate. The mucosal inoculation route is more efficient than the subcutaneous inoculation route; this can be explained by the possibility that the contaminated environment has a larger effect on renal carriage prevalence in a rat colony than biting.

### Other potential excretion routes

Using molecular methods, we investigated other potential excretion fluids that might enable *Leptospira* transmission, such as saliva, feces and BAL. Saliva has rarely been indicated as a contaminated fluid [36], but a few cases of bite transmission have been reported during the last century [37–39]. However, these have mainly been attributed to indirect urinary contamination of the mouth or wound. In our study, all saliva and salivary gland samples from infected rats tested negative for *Leptospira* by qPCR, even if those from infected rats that excreted a large number of leptospires in the urine and continued to groom themselves. The saliva was not contaminated, either by direct excretion from the salivary gland or by indirect contamination from urine during grooming.

We also assessed the feces, which have never been investigated. The idea to test the feces came from two assumptions: 1) if rats are contaminated by an infected environment, the ingested leptospires could be found in the feces; 2) because the gut is an immune escape site, leptospires could take advantage of this opportunity. All fecal samples from the infected rats tested negative for leptospires. The potentially ingested leptospires do not appear to survive in the rat digestive tract, most likely because they are destroyed by gastric acid [40].

Finally, we investigated the presence of leptospires in the BAL. All BAL samples from the infected rats tested negative for leptospires, but the pulmonary lesions observed in the perivascular region in our study may have resulted from an acute susceptibility to transitory leptospires. Pulmonary lesions with hemorrhages were previously described only in acute infection models [41,42], with the exception of infections in rat pups [33]. Hemorrhages in susceptible animal models are caused by vasculitis, which could be the cause of the perivascular lymphoid hypertrophy found in this study. Warthin-Starry staining and immunohistochemistry did not reveal leptospires in pulmonary sections, as observed previously [14]. However, leptospires are also rarely detected in the alveoli during acute infection [41]. Despite the negative BAL samples, leptospires appear to have a pathogenic effect on the rat lung.

### Conclusion

*Leptospira* infection in rats has identical asymptomatic forms regardless of the three inoculation routes (M, SC and IP) used. The antibody response in chronic infections was considerably lower than in acute infections, and antibody production had no relationship to the renal colonization rate. Therefore, natural routes of infection, such as mucosal and subcutaneous routes, affected both the time course of *Leptospira* dissemination in the body and the renal colonization associated with urinary excretion. The mucosal inoculation route was more efficient for renal colonization associated with *Leptospira* urinary excretion than the subcutaneous route. This is the first report of observations in the rat model using new inoculation routes (mucosal and subcutaneous) compared with the reference intraperitoneal inoculation route. Our investigation of other potential excretory fluids, such as saliva, feces and BAL, showed that these fluids do not appear to transmit *Leptospira*. We suggest using natural routes of *Leptospira* infection in future studies of rat models to investigate infection kinetics and renal colonization rates under natural conditions.

## Supporting Information

S1 Fig Hematoxylin-phloxine examination of the lungs and kidney of rats infected with *Leptospira*. A, Pulmonary sections obtained from one intraperitoneally infected rat showing a perivascular lymphoid hypertrophy (arrow). B, Renal sections obtained from one subcutaneously infected rat showing interstitial nephritis (arrow).
(TIF)

S1 Table The body weight gains of the rats from infected and control groups during the one-month study period. (DOCX)

S2 Table Renal colonization and urinary excretions of each infected rat on day 30. (DOCX)

## References

[1] Adler B, de la Pena Moctezuma A. Leptospira and leptospirosis. Vet Microbiol. 27 Janv 2010;140:287–96. 10.1016/j.vetmic.2009.03.012 19345023

[2] Picardeau M. Diagnosis and epidemiology of leptospirosis. Med Mal Infect. Janv 2013;43:1–9. 10.1016/j.medmal.2012.11.005 23337900

[3] Abela-Ridder B, Sikkema R, Hartskeerl RA. Estimating the burden of human leptospirosis. Int J Antimicrob Agents. 11 2010;36 Suppl 1:S5–7. 10.1016/j.ijantimicag.2010.06.012 20688484

[4] McBride AJA, Athanazio DA, Reis MG, Ko AI. Leptospirosis. Curr Opin Infect Dis. 10 2005;18(5):376–86. 16148523 10.1097/01.qco.0000178824.05715.2c

[5] Guerra MA. Leptospirosis. J Am Vet Med Assoc. 15 Févr 2009;234(4):472–8, 430. 10.2460/javma.234.4.472 19222355

[6] Vijayachari P, Sugunan AP, Shriram AN. Leptospirosis: an emerging global public health problem. J Biosci. 11 2008;33(4):557–69. 19208981 10.1007/s12038-008-0074-z

[7] Schuller S, Francey T, Hartmann K, Hugonnard M, Kohn B, Nally JE, et al European consensus statement on leptospirosis in dogs and cats. J Small Anim Pract. Mars 2015;56(3):159–79. 10.1111/jsap.12328 25754092

[8] Ido Y, Hoki R, Ito H, Wani H. THE RAT AS A CARRIER OF SPIROCHAETA ICTEROHAEMORRHAGIAE, THE CAUSATIVE AGENT OF WEIL’S DISEASE (SPIROCHAETOSIS ICTEROHAEMORRHAGICA). J Exp Med. 1 9 1917;26(3):341–53. 19868153 10.1084/jem.26.3.341 PMC2125787

[9] Bharti AR, Nally JE, Ricaldi JN, Matthias MA, Diaz MM, Lovett MA, et al Leptospirosis: a zoonotic disease of global importance. Lancet Infect Dis. Déc 2003;3:757–71. 14652202 10.1016/s1473-3099(03)00830-2

[10] Hartskeerl RA, Collares-Pereira M, Ellis WA. Emergence, control and re-emerging leptospirosis: dynamics of infection in the changing world. Clin Microbiol Infect. Avr 2011;17:494–501. 10.1111/j.1469-0691.2011.03474.x 21414083

[11] Dupouey J, Faucher B, Edouard S, Richet H, Kodjo A, Drancourt M, et al Human leptospirosis: an emerging risk in Europe? Comp Immunol Microbiol Infect Dis. Mars 2014;37(2):77–83. 10.1016/j.cimid.2013.12.002 24388481

[12] Watanabe T, Teske SS, Haas CN. Classic dose-response and time postinoculation models for leptospira. Risk Anal Off Publ Soc Risk Anal. Mars 2014;34(3):465–84. 10.1111/risa.12122 24117870

[13] Bonilla-Santiago R, Nally JE. Rat model of chronic leptospirosis. Curr Protoc Microbiol. Févr 2011;Chapter 12:Unit 12E.3. 10.1002/9780471729259.mc12e03s20 21400676

[14] Athanazio DA, Silva EF, Santos CS, Rocha GM, Vannier-Santos MA, McBride AJ, et al Rattus norvegicus as a model for persistent renal colonization by pathogenic Leptospira interrogans. Acta Trop. Févr 2008;105:176–80. 18093568 10.1016/j.actatropica.2007.10.012

[15] Thiermann AB. The Norway rat as a selective chronic carrier of Leptospira icterohaemorrhagiae. J Wildl Dis. Janv 1981;17(1):39–43. 7253100 10.7589/0090-3558-17.1.39

[16] Monahan AM, Callanan JJ, Nally JE. Proteomic analysis of Leptospira interrogans shed in urine of chronically infected hosts. Infect Immun. 11 2008;76(11):4952–8. 10.1128/IAI.00511-08 18765721 PMC2573331

[17] Nally JE, Chow E, Fishbein MC, Blanco DR, Lovett MA. Changes in lipopolysaccharide O antigen distinguish acute versus chronic Leptospira interrogans infections. Infect Immun. Juin 2005;73:3251–60. 15908349 10.1128/IAI.73.6.3251-3260.2005 PMC1111870

[18] Villanueva SYAM, Saito M, Baterna RA, Estrada CAM, Rivera AKB, Dato MC, et al Leptospira-rat-human relationship in Luzon, Philippines. Microbes Infect Inst Pasteur. 11 2014;16(11):902–10. 10.1016/j.micinf.2014.07.001 25048015

[19] Costa F, Wunder EA, De Oliveira D, Bisht V, Rodrigues G, Reis MG, et al Patterns in Leptospira Shedding in Norway Rats (Rattus norvegicus) from Brazilian Slum Communities at High Risk of Disease Transmission. PLoS Negl Trop Dis. Juin 2015;9(6):e0003819 10.1371/journal.pntd.0003819 26047009 PMC4457861

[20] Krojgaard LH, Villumsen S, Markussen MD, Jensen JS, Leirs H, Heiberg AC. High prevalence of Leptospira spp. in sewer rats (Rattus norvegicus). Epidemiol Infect. 11 2009;137:1586–92. 10.1017/S0950268809002647 19393116

[21] Himsworth CG, Bidulka J, Parsons KL, Feng AYT, Tang P, Jardine CM, et al Ecology of Leptospira interrogans in Norway rats (Rattus norvegicus) in an inner-city neighborhood of Vancouver, Canada. PLoS Negl Trop Dis. 2013;7(6):e2270 10.1371/journal.pntd.0002270 23818996 PMC3688548

[22] Zhang Y, Lou X-L, Yang H-L, Guo X-K, Zhang X-Y, He P, et al Establishment of a leptospirosis model in guinea pigs using an epicutaneous inoculations route. BMC Infect Dis. 2012;12:20 10.1186/1471-2334-12-20 22273178 PMC3329641

[23] Coutinho ML, Matsunaga J, Wang L-C, de la Peña Moctezuma A, Lewis MS, Babbitt JT, et al Kinetics of Leptospira interrogans infection in hamsters after intradermal and subcutaneous challenge. PLoS Negl Trop Dis. 11 2014;8(11):e3307 10.1371/journal.pntd.0003307 25411782 PMC4239013

[24] Lourdault K, Aviat F, Picardeau M. Use of quantitative real-time PCR for studying the dissemination of Leptospira interrogans in the guinea pig infection model of leptospirosis. J Med Microbiol. Mai 2009;58:648–55. 10.1099/jmm.0.008169-0 19369528

[25] Tucunduva de Faria M, Athanazio DA, Goncalves Ramos EA, Silva EF, Reis MG, Ko AI. Morphological alterations in the kidney of rats with natural and experimental Leptospira infection. J Comp Pathol. 11 2007;137:231–8. 17996544 10.1016/j.jcpa.2007.08.001

[26] Ko AI, Galvão Reis M, Ribeiro Dourado CM, Johnson WD, Riley LW. Urban epidemic of severe leptospirosis in Brazil. Salvador Leptospirosis Study Group. Lancet. 4 9 1999;354(9181):820–5. 10485724 10.1016/s0140-6736(99)80012-9

[27] WHO. Human Leptospirosis: Guidance for Diagnosis, Surveillance and Control. World Health Organization Geneva, Switzerland; 2003. 109 p.

[28] Merien F, Portnoi D, Bourhy P, Charavay F, Berlioz-Arthaud A, Baranton G. A rapid and quantitative method for the detection of Leptospira species in human leptospirosis. FEMS Microbiol Lett. 1 Août 2005;249(1):139–47. 16006065 10.1016/j.femsle.2005.06.011

[29] Bourhy P, Bremont S, Zinini F, Giry C, Picardeau M. Comparison of real-time PCR assays for detection of pathogenic Leptospira spp. in blood and identification of variations in target sequences. J Clin Microbiol. Juin 2011;49:2154–60. 10.1128/JCM.02452-10 21471336 PMC3122738

[30] Zuerner RL. Laboratory maintenance of pathogenic Leptospira. Curr Protoc Microbiol. 10 2005;Chapter 12:Unit 12E 1. 10.1002/9780471729259.mc12e01s00 18770554

[31] Faine S. Silver Staining of Spirochaetes in Single Tissue Sections. J Clin Pathol. Mai 1965;18:381–2. 14304261 10.1136/jcp.18.3.381 PMC472950

[32] Fornazari F, da Silva RC, Richini-Pereira VB, Beserra HEO, Luvizotto MCR, Langoni H. Comparison of conventional PCR, quantitative PCR, bacteriological culture and the Warthin Starry technique to detect Leptospira spp. in kidney and liver samples from naturally infected sheep from Brazil. J Microbiol Methods. 9 2012;90(3):321–6. 10.1016/j.mimet.2012.06.005 22713608

[33] Muslich LT, Villanueva SYAM, Amran MY, Segawa T, Saito M, Yoshida S. Characterization of Leptospira infection in suckling and weaning rat pups. Comp Immunol Microbiol Infect Dis. Févr 2015;38:47–55. 10.1016/j.cimid.2014.11.001 25605653

[34] Agudelo-Florez P, Londono AF, Quiroz VH, Angel JC, Moreno N, Loaiza ET, et al Prevalence of Leptospira spp. in urban rodents from a groceries trade center of Medellin, Colombia. Am J Trop Med Hyg. 11 2009;81:906–10. 10.4269/ajtmh.2009.09-0195 19861630

[35] Paixao Mdos S, Alves-Martin MF, Tenorio Mda S, Starke-Buzetti WA, Alves ML, da Silva DT, et al Serology, isolation, and molecular detection of Leptospira spp. from the tissues and blood of rats captured in a wild animal preservation centre in Brazil. Prev Vet Med. 1 Juill 2014;115:69–73. 10.1016/j.prevetmed.2014.03.016 24703251

[36] Babudieri B. Animal reservoirs of leptospires. Ann N Y Acad Sci. 3 Juin 1958;70(3):393–413. 13559904 10.1111/j.1749-6632.1958.tb35398.x

[37] Gollop JH, Katz AR, Rudoy RC, Sasaki DM. Rat-bite leptospirosis. West J Med. Juill 1993;159(1):76–7. 8351916 PMC1022170

[38] Luzzi GA, Milne LM, Waitkins SA. Rat-bite acquired leptospirosis. J Infect. Juill 1987;15(1):57–60. 3668265 10.1016/s0163-4453(87)91451-4

[39] Roczek A, Forster C, Raschel H, Hörmansdorfer S, Bogner K-H, Hafner-Marx A, et al Severe course of rat bite-associated Weil’s disease in a patient diagnosed with a new Leptospira-specific real-time quantitative LUX-PCR. J Med Microbiol. Mai 2008;57(Pt 5):658–63. 10.1099/jmm.0.47677-0 18436602

[40] Asoh T, Saito M, Villanueva SYAM, Kanemaru T, Gloriani N, Yoshida S. Natural defense by saliva and mucosa against oral infection by Leptospira. Can J Microbiol. Juin 2014;60(6):383–9. 10.1139/cjm-2014-0016 24861456

[41] Nally JE, Chantranuwat C, Wu XY, Fishbein MC, Pereira MM, Da Silva JJ, et al Alveolar septal deposition of immunoglobulin and complement parallels pulmonary hemorrhage in a guinea pig model of severe pulmonary leptospirosis. Am J Pathol. Mars 2004;164:1115–27. 14982864 10.1016/S0002-9440(10)63198-7 PMC1614724

[42] Pereira MM, Da Silva JJP, Pinto MA, Da Silva MF, Machado MP, Lenzi HL, et al Experimental leptospirosis in marmoset monkeys (Callithrix jacchus): a new model for studies of severe pulmonary leptospirosis. Am J Trop Med Hyg. Janv 2005;72(1):13–20. 15728860

