# Supplementary figures and images for: Comparison of Mucosal, Subcutaneous and Intraperitoneal Routes of Rat Leptospira Infection
Source: PLoS Negl Trop Dis. 2016 Mar 31;10(3):e0004569. doi: 10.1371/journal.pntd.0004569 (PMC4816568; doi:10.1371/journal.pntd.0004569)

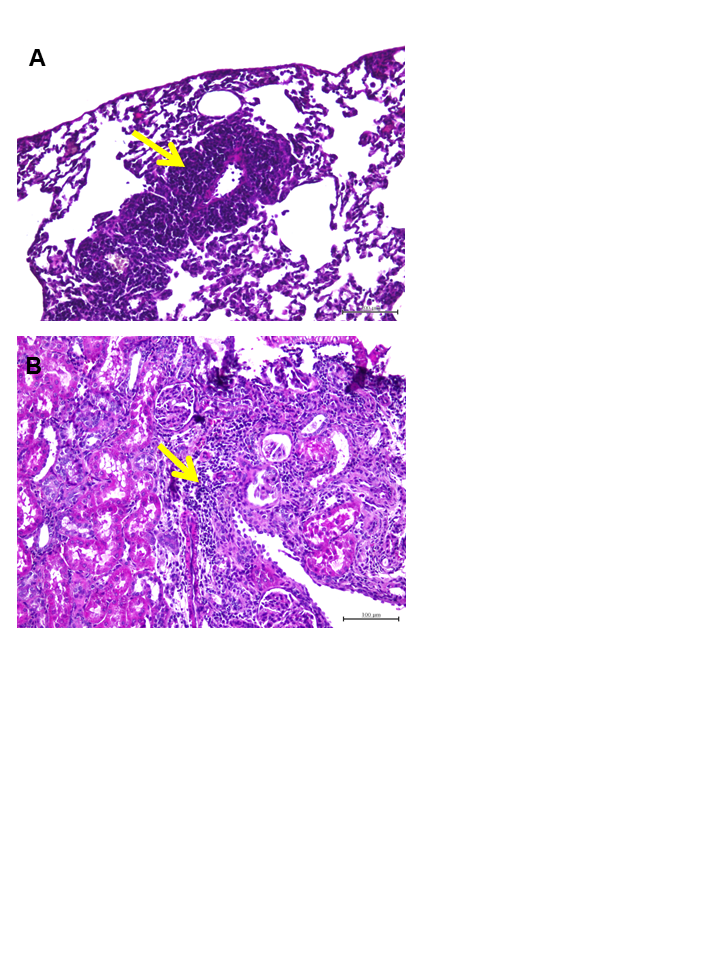

Supplement: S1 Fig — A, Pulmonary sections obtained from one intraperitoneally infected rat showing a perivascular lymphoid hypertrophy (arrow). B, Renal sections obtained from one subcutaneously infected rat showing interstitial nephritis (arrow). (TIF) [file pntd.0004569.s001.tif]
