# Supplementary material for: Comparison of Mucosal, Subcutaneous and Intraperitoneal Routes of Rat Leptospira Infection
Source: PLoS Negl Trop Dis. 2016 Mar 31;10(3):e0004569. doi: 10.1371/journal.pntd.0004569 (PMC4816568; doi:10.1371/journal.pntd.0004569)
Supplement: S1 Table — (DOCX) [file pntd.0004569.s002.docx]

**S1 Table: The body weight gains of the rats from infected and control groups during the one-month study period**

| **Inoculation route** | **Infected group*** | **Control group*** |
| --- | --- | --- |
| **Intraperitoneal** | 155 | 129 |
| **Subcutaneous** | 150 | 162 |
| **Mucosal** | 147 | 165 |

* The mean of weight gains are indicated in gram.
