# Supplementary material for: Comparison of Mucosal, Subcutaneous and Intraperitoneal Routes of Rat Leptospira Infection
Source: PLoS Negl Trop Dis. 2016 Mar 31;10(3):e0004569. doi: 10.1371/journal.pntd.0004569 (PMC4816568; doi:10.1371/journal.pntd.0004569)
Supplement: S2 Table — (DOCX) [file pntd.0004569.s003.docx]

**S2 Table: Renal colonization and urinary excretions of each infected rat on day 30.**

| **Infected group** |  | **Rat 1** | **Rat 2** | **Rat 3** | **Rat 4** | **Rat 5** | **Rat 6** | **Rat 7** | **Rat 8** |
| --- | --- | --- | --- | --- | --- | --- | --- | --- | --- |
| **Intraperitoneal** | **Urine qPCR^a^** | 937 | 4538 | 664 | 4381 | 1724 | 2591 | 1140 | 297 |
|  | **Kidney qPCR^a^** | 2 | 21 | 8 | 93 | 130 | 12 | 14 | 60 |
|  | **Kidney isolation^b^** | + | + | + | + | + | + | + | - |
| **Subcutaneous** | **Urine qPCR^a^** | 0 | 0 | 0 | 0 | 0 | 0 | 0 | 0 |
|  | **Kidney qPCR^a^** | 0 | 0 | 0 | 0 | 0 | 0 | 0 | 0 |
|  | **Kidney isolation^b^** | - | - | + | - | - | - | - | - |
| **Mucosal** | **Urine qPCR^a^** | 0 | 354 | 693 | 0 | 715 | 454 | 120 | 0 |
|  | **Kidney qPCR^a^** | 0 | 0 | 12 | 0 | 2 | 0 | 6 | 0 |
|  | **Kidney isolation^b^** | - | - | + | - | + | + | + | - |

**^a^** The concentrations of DNA of the positive samples are indicated in genomic copies/µL of DNA extract (1 µL of DNA extract is equal to 1 µL of urine or 0.1 mg of kidney).

**^b^** +: the renal isolation is positive; -: the renal isolation is negative.
